# Assessment of Public Knowledge and Perceptions Toward Radiation Exposure Risks in Saudi Arabia: A Survey Study

**DOI:** 10.7759/cureus.80351

**Published:** 2025-03-10

**Authors:** Abdullah A Alburayh, Mead Alosaimi, Hend Alshumiesy, Aghnar T Alzahrani, Ali S Alkhars, Delal M Doaib, Mohammed H Alsaleh, Abdullah Albakri, Noora Abdulrahman Alrajhi, Beshair Almansour, Sumaeah Alghamdi, Salem Hamad Aldosari, Ayman S Alhasan

**Affiliations:** 1 Medicine, King Faisal University, Hofuf, SAU; 2 Medicine, Taif University, Taif, SAU; 3 Pediatric Surgery, Security Forces Hospital, Riyadh, SAU; 4 Medicine and Surgery, Al Baha University, Al Bahah, SAU; 5 Surgery, King Abdulaziz University, Jeddah, SAU; 6 Diagnostic Radiology, Al-Mouwasat Hospital, Dammam, SAU; 7 Diagnostic Radiology, King Saud Hospital, Unaizah, SAU; 8 College of Medicine, Imam Mohammad Ibn Saud Islamic University, Riyadh, SAU; 9 General Practice, King Khalid Primary Healthcare Center, Unaizah, SAU; 10 Medicine, Faculty of Medicine, Al Baha University, Al Bahah, SAU; 11 Radiology, Wadi Al-Dawasir General Hospital, Ministry of Health, Wadi Al-Dawasir, SAU; 12 Radiology, College of Medicine, Taibah University, Madinah, SAU

**Keywords:** kingdom of saudi arabia (ksa), pediatric radiation exposure, public knowledge, radiation, radiation protection measures, radiography

## Abstract

Introduction

Radiation exposure poses potential health risks, yet public awareness regarding its sources, effects, and safety measures remains inconsistent. Understanding the level of knowledge and perceptions about radiation risks is essential for developing effective educational strategies. This study aimed to assess public knowledge, perceptions, and attitudes toward radiation exposure risks in Saudi Arabia, focusing on awareness of ionizing radiation sources, safety measures, and information-seeking behaviors.

Materials and methods

A cross-sectional survey was conducted among Saudi residents aged 18 years and above using an online questionnaire distributed through digital platforms. A convenience sampling method was used to recruit participants, and data were analyzed using descriptive statistics, chi-square tests, and logistic regression models in SPSS version 27 (IBM Corp., Armonk, NY). The study examined associations between demographic factors and levels of knowledge and perception regarding radiation exposure risks.

Results

Over two-thirds (67.6%) were unaware of natural sources of ionizing radiation, and 41.5% incorrectly believed that chest X-rays and CT scans deliver equal radiation doses. The most common imaging procedures experienced were simple X-rays (28.9%) and dental X-rays (25.7%). More than half (53.1%) reported concerns during radiological tests, and 58.3% believed these procedures pose health hazards. Receiving prior information on radiation risks strongly predicted higher awareness (p < 0.001), while males and married individuals were less likely to have positive perceptions (p < 0.05).

Conclusion

The study identified significant gaps in public knowledge and perception toward radiation exposure risks. Receiving information on the topic was a key predictor of increased awareness, while gender and marital status influenced perceptions, with males and married participants being less likely to hold positive views. These findings emphasize the need for targeted educational interventions and tailored communication strategies to address knowledge gaps, dispel misconceptions, change attitudes, and foster patient trust regarding radiation exposure risks and radiological procedures.

## Introduction

Radiation exposure in our environment can be classified into ionizing radiation and non-ionizing radiation. Ionizing radiation can pass through various materials and penetrate the human body, where it can be absorbed by tissues. Exposure to high levels of ionizing radiation has the potential to cause harmful effects on individuals [1].

Radiation exposure to the central nervous system is associated with various health risks and potential complications such as hyperprolactinemia or gonadotropin deficiency, particularly when the hypothalamic-pituitary axis is within the radiation field. Similarly, direct irradiation of the testis can affect the germinal epithelium, leading to aspermia at doses exceeding 0.35 Gy. It is important to note that lower doses may cause reversible aspermia, while higher doses can prolong the recovery period. However, doses exceeding 2 Gy may result in permanent aspermia [2].

Radiation exposure and environmental factors contribute to the rising incidences of thyroid cancers globally. In 2008, the estimated age-standardized incidence rates for thyroid cancer worldwide were 4.7 per 100,000 women and 1.5 per 100,000 men [3]. This increase in incidence can be attributed to various environmental factors, including changes in population exposure to ionizing radiation from fallout, diagnostic tests, and treatments for both benign and malignant conditions. The symptoms and severity of the conditions occasioned by the radiation exposure vary depending on the duration and the dose of the exposure [4]. The radiation exposure during childhood can potentially affect growth and development, including impairments in cognitive function, learning difficulties, and a higher risk of developing certain genetic disorders [4].

The fundamental principles of radiation protection encompass justification, enhancing protection and safety measures, and implementing dose limits. In medical practices involving ionizing radiation, achieving the most precise and dependable results with minimal radiation exposure is only attainable by adhering to these core principles. The level of awareness among healthcare professionals regarding the detrimental effects of radiation on human health directly influences their ability to uphold these principles effectively [5]. While previous research has noted that knowledge of radiation exposure and its potential risks influence adherence to the safety measures, it is crucial to understand public awareness and perception toward radiation exposure risk in Saudi Arabia. This is fundamental, considering the unique healthcare, cultural, environmental, and regulatory aspects in the country that may influence how the public perceives and responds to radiation exposure risks.

Nurses play a central role in healthcare facilities by providing essential care and support to patients. There was a study conducted among nursing students, and the results showed more than half (52%) of the final-year nursing students did not participate in any radiation protection courses, as revealed by the survey. Furthermore, the findings from the survey suggest a significant lack of awareness regarding basic radiation protection knowledge among final-year nursing students in Fatima College of Health Sciences (FCHS) campuses, with less than 80% demonstrating adequate understanding. The results indicate a deficiency in knowledge and a negative attitude toward radiation hazards and radiation protection among the final-year nursing students in the FCHS [6].

In a study conducted among physicians to assess the level of knowledge and awareness regarding the potential hazards of radiological examinations on their patients' health, out of the 466 participants, 73% demonstrated significant knowledge gaps. These gaps were evident in various aspects, such as the classification of mammography as ionizing radiation, with 51% of respondents unable to correctly identify it as such. Additionally, 69.3% of participants were unaware of the recommended annual dose limit for radiation workers' whole-body exposure. The overall knowledge score ranged from 0% to 16.5%, with an average score of 5.3%. Notably, surgeons and orthopedists had particularly low scores in terms of their knowledge of the subject. These findings underscore the need for further education and awareness among physicians regarding the hazards of radiological examinations on both their own health and that of their patients [7].

In a related context, a study assessing patient awareness of medical radiation exposure revealed similar gaps in knowledge. More than half (56.4%) of the 737 respondents were unaware of which medical imaging modalities use ionizing radiation, and 74.7% had never discussed the potential risks of medical radiological procedures with healthcare professionals. Furthermore, 70.1% were unaware of the healthcare professionals qualified to discuss the use of ionizing radiation. Despite these gaps, 84.7% of participants believed that radiation dose information should be included in their medical reports. In contrast, a study conducted within the Diagnostic Radiology Department, which evaluated the knowledge and practices of healthcare workers, demonstrated a more positive level of understanding. Over 90% of the 93 participants showed good knowledge of radiation hazards, exposure doses, and monitoring, while 87% exhibited a solid grasp of personal protective equipment (PPE) usage. Moreover, 74.2% adhered to PPE practices, and the presence of lead material in unit construction, as well as a radiation safety officer, ensured that proper safety protocols were consistently followed [8,9]. This study will seek to identify the misconception about radiation exposure risks, major sources of radiation, safety measures, and the difference in radiation awareness across the demographic.

## Materials and methods

Study design

A cross-sectional study was done to assess the level of knowledge and perception regarding radiation among the Saudi population via an online survey from December 2023 to February 2024. The study was approved by the Ethics Committee of King Faisal University in Al-Ahsa (KFU-REC-2023-DEC-ETHICS1779).

Sample size and sampling technique

The sample size was determined using a 95% confidence level and a 5% margin of error, calculated with the formula: $n = P (1-P) * \frac{Z_a^2}{d^2}$. This resulted in an estimated sample size of 385. However, we increased the sample to 429 to improve reliability. The non-probability convenience sampling technique was used to select participants considering their willingness and availability to participate. While the results from this method may be biased and limit generalizability, it is quick, provides easy access to participants, and is considerably cost-effective.

Inclusion and exclusion criteria

The study targeted adult individuals, aged 18 years and older, regardless of gender. Inclusion criteria required participants to be residents of Saudi Arabia, willing to participate, have access to the internet or social media, and at least above 18 years old. Exclusion criteria included non-residents, individuals unwilling to participate, individuals without access to the internet or social media, and those below 18 years old.

Data collection process

After receiving approval from the Institutional Ethics Committee of King Faisal University, data collectors used social media and emails to collect data from all regions of Saudi Arabia using a structured questionnaire in electronic format modified from previous studies [10,11]. The questionnaire was created using Google Forms (Google, Mountain View, CA) and distributed to participants via social media platforms, including WhatsApp, Facebook, and Instagram (Meta, Menlo Park, CA). This allowed both data collectors and participants to easily navigate the data collection process.

Confidentiality and protection of participants

Informed consent was obtained from all participants, who were made aware of their right to refuse participation or withdraw at any time. Additionally, the study’s objectives, data collection methods, and safety assurances were clearly explained. To maintain confidentiality and anonymity, participants’ names were not disclosed on surveys or research reports, and the collected data were kept confidential. The identities of the participants were only accessible to the current authors.

Data analytics

SPSS for Windows version 28 (IBM Corp., Armonk, NY) was used to code and analyze the data. Categorical variables are summarized as counts and proportions, while continuous variables, such as age, are expressed as means with standard deviations. The comparison of qualitative data among the groups was done using the chi-square (χ2) test, with statistical significance set at p-value < 0.05. Univariate logistic regression analysis was used to identify the independent factors that predict high awareness and knowledge, as well as the factors that predict positive perceptions regarding radiation exposure risks among participants.

## Results

This study included 429 participants assessed for radiation exposure awareness among the Saudi population (Table 1). In terms of gender, 64.1% were female, and 35.9% were male. Regarding age distribution, the majority (66.4%) were ≤29 years old, with smaller percentages in higher age brackets. Saudi nationality dominated at 93.0%. Most participants were single (65.3%), and the highest educational attainment of participants was university level (79.0%). Regionally, the Eastern region had the highest representation (32.9%). The diverse occupation categories included students (47.3%), reflecting the study's broad demographic scope.

**Table 1:** Sociodemographic and other parameters of participants presented in frequencies (n) and percentage (%).

		Frequency (n = 429)	Percent (out of 100%)
Gender	Female	275	64.1%
	Male	154	35.9%
Age	≤29 years	285	66.4%
	30-39 years	33	7.7%
	40-49 years	44	10.3%
	≥50 years	67	15.6%
Nationality	Non-Saudi	30	7.0%
	Saudi	399	93.0%
Marital status	Single	280	65.3%
	Married	139	32.4%
	Widow/divorced	10	2.3%
Region	Northern	20	4.7%
	Southern	27	6.3%
	Central	113	26.3%
	Western	128	29.8%
	Eastern	141	32.9%
Education	Below secondary	5	1.2%
	Secondary	85	19.8%
	University‎	339	79.0%
Occupation	Unemployed/retired	62	14.5%
	Part-time employee	15	3.5%
	Housewife	29	6.8%
	Full-time employee	120	28.0%
	Student	203	47.3%

**Figure 1:**
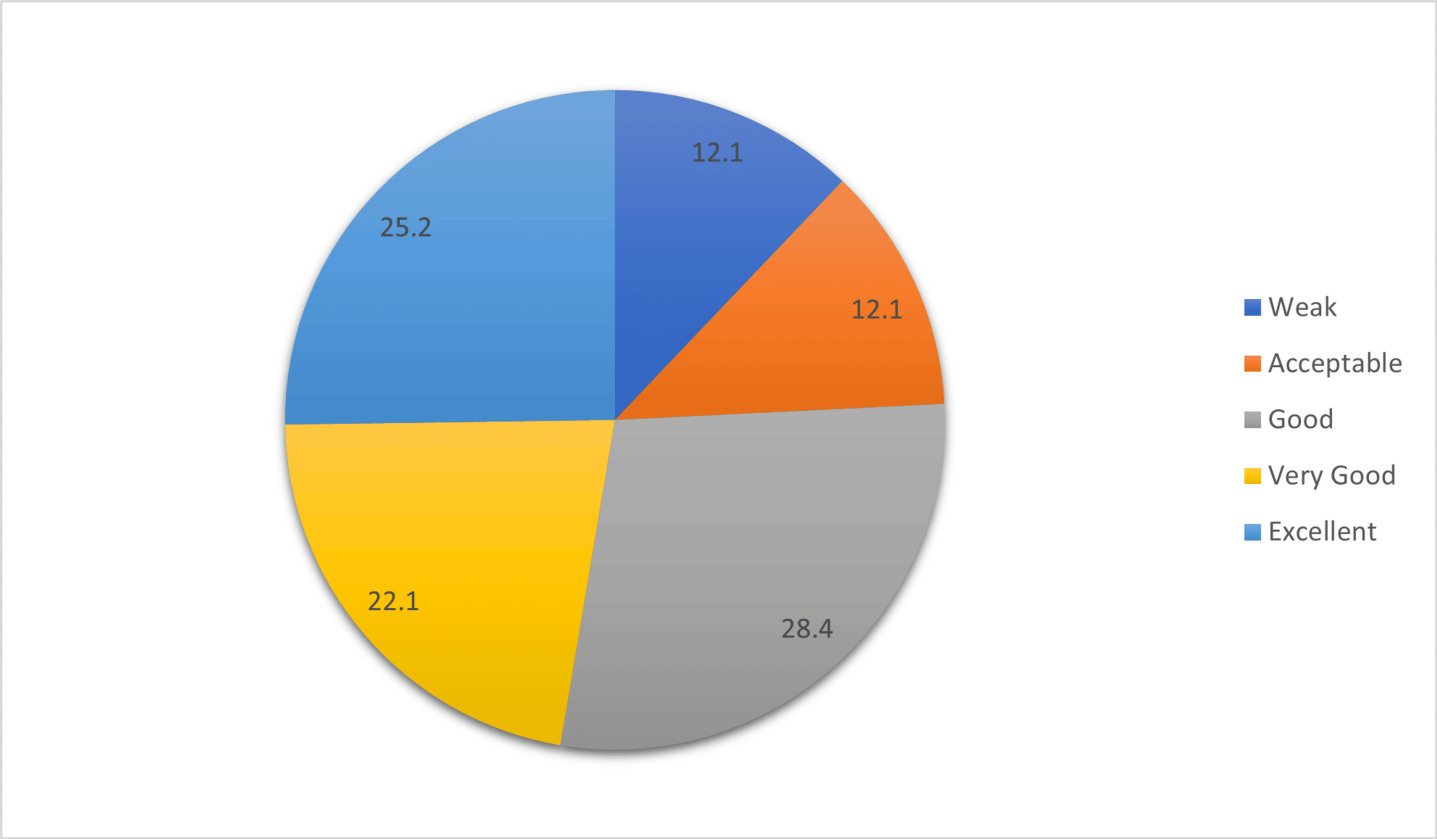
Participants' rating of knowledge about radiation exposure risks according to their own perception.

Figure 1 shows participants' self-perceived knowledge levels on radiation exposure risks. Notably, 8.4% considered their understanding as "Good," and 25.2% rated it as "Excellent." About 22.1% felt "Very Good," while 12.1% found their knowledge "Weak," and another 12.1% deemed it "Acceptable."

**Figure 2:**
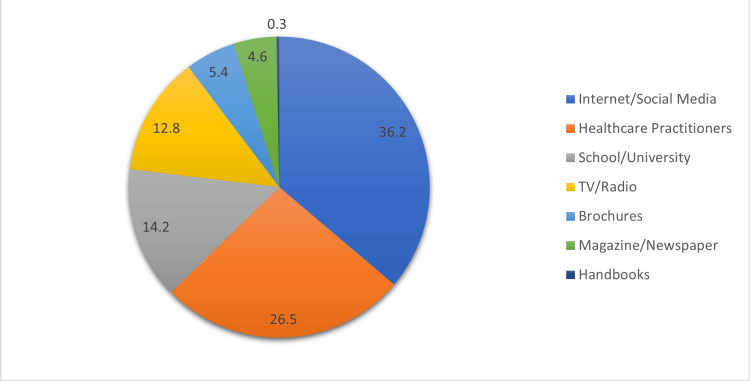
Major sources through which the Saudi public receives information about radiation risks.

Figure 2 shows the primary channels for information dissemination about radiation exposure risks among the Saudi public. Notably, 36.2% rely on the internet and social media. Healthcare practitioners contribute significantly at 26.5%, underlining the role of medical professionals in education. Schools/universities play a substantial role, contributing to 14.2%, while traditional media like TV/radio (12.8%) and print materials such as brochures (5.4%) also contribute, albeit to a lesser extent. Handbooks make a minimal contribution at 0.3%.

Table 2 presents an assessment of public knowledge, knowledge gaps, and misconceptions regarding radiation exposure risks among participants. Most participants (62.7%) had received information about the risks of radiological imaging, with an overwhelming majority (87.1%) preferring to receive information from radiologists. A significant proportion (67.6%) was unaware of natural sources of ionizing radiation. Notable knowledge gaps were identified, including a considerable minority (41.5%) incorrectly reporting that chest X-rays and CT scans have equal radiation doses. More than half of the participants (54.3%) were unaware that certain tests emit radiation. Additionally, most participants (60.4%) were uncertain about radiation dose differences in abdominal CT scans based on patient size. The majority (69.9%) perceived radiological tests as dangerous, and a notable proportion (53.4%) believed that these tests are riskier for children.

**Table 2:** Assessment of the level of public knowledge, knowledge gaps, and misconceptions regarding radiation exposure risks. Public knowledge, knowledge gaps, and misconceptions regarding radiation exposure risks are presented in frequencies (n) and percentages (%).

		Frequency (n = 429)	Percent (out of 100%)
Have you ever received information about the risks associated with the use of radiological imaging (radiography) for medical examinations?	No	160	37.3%
	Yes	269	62.7%
From which healthcare professional would you prefer to receive information about the risks associated with ionizing radiation?	Radiologist	374	87.1%
	Radiographer	223	51.9%
	Medical physicist	112	26.1%
	General practitioner	112	26.1%
Are you aware of the natural sources of ionizing radiation to which we are all exposed?	No	290	67.6%
	Yes	139	32.4%
Which of these radiological examinations involve exposure to ionizing radiation?	CT	187	43.5%
	MRI	105	24.4%
	Mammogram	88	20.5%
	Ultrasound	96	22.3%
Which imaging test exposes a person to the highest radiation dose?	Chest X-ray	93	21.7%
	CT of the chest	158	36.8%
	Both have equal	178	41.5%
A person emits radiation after which of these tests?	CT with contrast	22	5.1%
	Contrast USG	15	3.5%
	Scintigraphy (nuclear rays)	97	22.6%
	All of the above	47	11.0%
	Don't know	233	54.3%
	None of the above	15	3.5%
For an abdominal CT scan, how does the radiation dose compare between a thinner patient (60 kg) and a larger patient (100 kg)?	Equal	54	12.6%
	Higher in heavier patients	84	19.6%
	Higher in the lighter patient	32	7.5%
	I don't know	259	60.4%
How dangerous do you think it is to undergo radiological tests using ionizing radiation?	Not dangerous	57	13.3%
	Kind of dangerous	300	69.9%
	Very dangerous	72	16.8%
For which demographic is it riskier to undergo a radiological test using ionizing radiation?	A child	229	53.4%
	25-year-old man	4	0.9%
	25-year-old woman	16	3.7%
	Middle-aged adult	22	5.1%
	Elderly	33	7.7%
	No difference (similar risk)	125	29.1%

**Figure 3:**
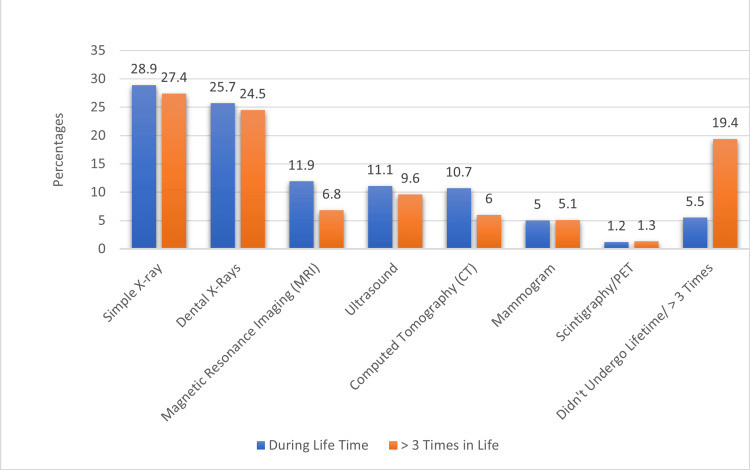
Evaluation of radiological procedures that participants have undergone during their lifetime or more than three times in their life. CT: computed tomography; MRI: magnetic resonance imaging; PET: positron emission tomography.

Figure 3 shows an examination of radiological procedures experienced by participants during their lifetime or more than three times. Among lifetime occurrences, simple X-rays (28.9%) and dental X-rays (25.7%) were the most common, followed by magnetic resonance imaging (MRI) and ultrasound, each around 11%. Mammograms accounted for only 5%, while scintigraphy/PET procedures were less common at 1.2%. Notably, about 5.5% of participants had not undergone any radiological procedures in their lifetime. For procedures experienced more than three times, dental X-rays (24.5%) closely followed simple X-rays (27.4%), while those who had not undergone such procedures decreased to 19.4%.

Table 3 provides a comprehensive overview of the public's perceptions of radiation exposure risks during medical procedures. Notably, more than half (53.1%) expressed concern during radiological tests, while 58.7% were satisfied with a diagnosis that did not require further radiological investigation. A significant portion (57.3%) indicated that they would not avoid radiological tests if they were tolerable, reflecting a willingness to undergo such procedures. Concerns about hazards and side effects were acknowledged by the majority (58.3%). Nearly half of the participants (49.4%) reported feeling worried due to the absence of radiologists in the room, and about one-quarter (25.2%) felt anxious due to the radiologists' instructions.

**Table 3:** Assessment of the level of public perceptions regarding radiation exposure risks. Public perceptions regarding radiation exposure risks are presented in frequencies (n) and percentages (%).

		Disagree	Neutral	Agree
Do you worry during radiological test procedures?	N	165	36	228
	%	38.5	8.4	53.1
Would you avoid radiological tests if you could tolerate the disease?	N	246	82	101
	%	57.3	19.1	23.6
Are you satisfied with a diagnosis that doesn't require further investigation through radiological tests?	N	103	74	252
	%	24.0	17.3	58.7
Do you believe that radiology tests cause hazards and side effects?	N	80	99	250
	%	18.6	23.1	58.3
Does the absence of radiologists in the same room during procedures make you feel worried?	N	168	49	212
	%	39.2	11.4	49.4
Do instructions given by the radiologist make you feel anxious?	N	258	63	108
	%	60.1	14.7	25.2

The logistic regression results (Table 4) revealed that participants who received information about radiological risks (adjusted odds ratio (AOR) = 8.176; 95% CI = 4.062-16.458; p < 0.001) were significantly more likely to have high awareness and knowledge regarding radiation exposure risks. Additionally, being married (AOR = 1.917; 95% CI = 0.831-4.426; p = 0.128) and being a student (AOR = 1.169; 95% CI = 0.918-1.489; p = 0.206) showed positive associations, although these results were not statistically significant. Conversely, factors such as age, gender, nationality, residence area, academic level, and employment status did not have a significant influence on high awareness.

**Table 4:** Univariate logistic regression for the relationship between factors and high awareness and knowledge regarding radiation exposure risks among participants. AOR: adjusted odds ratio; CI: confidence interval. * Significant at p < 0.05 level.

	Sig.	AOR	95% CI	
			Lower	Upper
Age	.235	1.283	.851	1.933
Gender (male)	.136	.614	.323	1.166
Marital status (married)	.128	1.917	.830	4.426
Nationality (Saudi)	.844	1.130	.335	3.813
Residence area (eastern)	.672	.937	.694	1.265
Academic level (university)	.382	.698	.312	1.562
Employment status (student)	.206	1.169	.918	1.489
Received information about radiological risk (yes)	.000*	8.176	4.062	16.458

The logistic regression results (Table 5) showed that older participants (AOR = 1.518; 95% CI = 1.173-1.964; p < 0.001) were significantly more likely to have positive perceptions of radiation exposure risks, suggesting that older individuals are more likely to view radiological examinations favorably. In contrast, male participants (AOR = 0.607; 95% CI = 0.401-0.918; p = 0.018) and married participants (AOR = 0.590; 95% CI = 0.351-0.992; p = 0.047) were significantly less likely to hold positive perceptions regarding radiation exposure risks. Conversely, factors such as having a university education (AOR = 1.578; 95% CI = 0.988-2.520; p = 0.056) and being a student (AOR = 1.069; 95% CI = 0.901-1.270; p = 0.443) showed positive associations, though these results were not statistically significant.

**Table 5:** Different factors of positive perceptions regarding radiation exposure among participants (logistic regression model). AOR: adjusted odds ratio; CI: confidence interval. * Significant at p < 0.05 level.

	Sig.	AOR	95% CI	
			Lower	Upper
Age	.001*	1.518	1.173	1.964
Gender (male)	.018*	.607	.401	.918
Marital status (married)	.047*	.590	.351	.992
Nationality (Saudi)	.988	1.006	.449	2.257
Residence area (eastern)	.337	1.094	.910	1.316
Academic level (university)	.056	1.578	.988	2.520
Employment status (student)	.443	1.069	.901	1.270
Received information about radiological risk (yes)	.742	.934	.623	1.401

## Discussion

Radiation exposure risks constitute a significant public health concern, with human activities, particularly those involving medical procedures, serving as potential carcinogenic sources that affect millions globally [12,13]. Exposure to radiation has been associated with a range of adverse health outcomes, including permanent aspermia, thyroid cancer, cognitive impairments, and an increased susceptibility to genetic disorders [14-16]. These risks underscore the critical need for a comprehensive understanding of, and adherence to, radiation protection principles to mitigate potential harm. This study aims to evaluate public awareness and perceptions regarding radiation exposure risks in Saudi Arabia, given the potential occupational hazards and public health implications associated with such exposures.

Our demographic analysis revealed a predominantly young, single, and highly educated participant group, reflecting the evolving landscape of the Saudi population. Most participants were under 29 years of age, with 339 individuals (79.0%) possessing a university-level education. This trend is consistent with the global shift toward higher education attainment among younger populations. The sample was predominantly female (64.1%, n = 275), which may suggest gender-based differences in healthcare-seeking behaviors, particularly regarding concerns about radiation exposure to family members and children. This finding aligns with the research by Rasmussen et al. (2020), which indicates a heightened concern about radiation exposure among women and parents with children at home [17].

Participants demonstrated varying levels of self-perceived knowledge regarding radiation exposure. Specifically, 25.2% (n = 108) rated their understanding as "Excellent," while 28.4% (n = 122) considered it "Good." These findings provide valuable insight into the public's confidence in their understanding of radiation-related concepts. In line with this, Qari et al. (2023) reported that patients often lack awareness of ionizing radiation exposure equivalencies between different imaging modalities [18]. The reliance on the internet and social media as primary sources of information (36.2%, n = 155) underscores the growing influence of digital platforms in disseminating health-related knowledge. This trend is consistent with the findings of Hijlis et al. (2022), who highlighted that social media can play a significant role in enhancing radiation safety awareness [19]. Healthcare practitioners remained influential, with 26.5% (n = 114) of participants citing them as a key information source, aligning with existing literature that emphasizes the crucial role of medical professionals in public health education. Ribeiro et al. (2020) demonstrated that effective communication between patients and healthcare professionals regarding ionizing radiation enhances patient knowledge, awareness, and confidence in treatment decisions [20]. These findings collectively highlight the multifaceted nature of public education on radiation safety, with digital platforms and healthcare professionals both playing vital roles in shaping public understanding.

Our study identified significant gaps and misconceptions in public awareness of radiation exposure. While 269 participants (62.7%) reported having received information about radiological risks, a concerning 290 participants (67.6%) were unaware of natural sources of ionizing radiation. A prevalent misconception was observed regarding the equivalency of radiation doses from chest X-rays and CT scans, with 41.5% (n = 178) of participants incorrectly believing the doses to be identical. This highlights a critical need for targeted educational efforts focused on specific imaging modalities. In support of this, Sweetman et al. (2020) found that patients often underestimate the cancer risk associated with CT scans and are unable to accurately compare risks between different imaging techniques [21]. Additionally, 233 participants (54.3%) expressed uncertainty regarding which tests emit radiation, indicating a general lack of clarity about potential exposure sources. This uncertainty was further reflected in the finding that 60.4% (n = 259) of participants were unsure about radiation dose differences in abdominal CT scans based on patient size, underscoring the need for targeted educational interventions to address these gaps in knowledge.

This study provides valuable insights into participants' experiences with radiological procedures. Simple X-rays and dental X-rays were prevalent, with a significant proportion of participants undergoing these procedures more than three times during their lifetime, indicating a substantial burden of X-ray use in medical investigations. In line with these findings, Hubail (2023) reported that most patients presenting with symptoms indicative of acute appendicitis underwent plain X-rays of the chest, abdomen, or both [22]. However, 19.4% (n = 83) of participants reported having undergone no procedure more than three times, highlighting the diverse range of experiences within the population. These results underscore the importance of tailoring healthcare communication to accommodate the varied radiological histories of patients.

The notable proportion of patients expressing concern (53.1%, n = 228) highlights the emotional impact associated with these procedures, a factor that healthcare providers must address to ensure effective patient communication. As demonstrated by Rockall et al. (2022), communication with patients, their families, and caregivers must be empathetic, recognize the patient's priorities, and actively involve them in the decision-making process [23]. In contrast, 58.7% (n = 252) of patients expressed satisfaction with a diagnosis that did not necessitate further radiological investigation, suggesting a degree of trust in alternative diagnostic approaches. However, the concern regarding potential risks and side effects (58.3%, n = 250) underscores the necessity for clear and transparent communication to alleviate such apprehensions. The absence of radiologists during procedures was found to significantly contribute to patient worry (49.4%, n = 212), corroborating previous findings that patients undergoing therapeutic procedures without a radiologist present often experience increased anxiety [24]. This emphasizes the critical importance of patient-centered care in mitigating distress and fostering trust.

The logistic regression analysis revealed that receiving information about radiological risks (AOR = 8.176; 95% CI = 4.062-16.458; p < 0.001) was a significant predictor of heightened awareness regarding radiation exposure and the adoption of protective behaviors. This finding aligns with Park et al. (2021), who demonstrated that greater knowledge and awareness of radiation protection are associated with increased protective behaviors (r = 0.37, p < 0.001) [25]. Although marital status and student status were positively associated with awareness and protective behaviors, these associations did not reach statistical significance. Age showed a positive correlation with favorable perceptions, whereas gender and marital status were inversely associated with positive perceptions. While descriptive analysis indicated general knowledge gaps, regression analysis identified significant predictors of awareness, such as prior exposure to radiation-related information. Additionally, certain demographic variables (e.g., gender and marital status) showed different associations with perception levels when analyzed through regression models compared to simple frequency distributions. These discrepancies highlight the complexity of the public understanding of radiation risks and the need for multi-method analytical approaches to capture both general patterns and underlying influences.

Limitations and future directions

The limitations of this study were considered while interpreting the findings. The cross-sectional design allows for the identification of associations between study attributes but cannot establish causal relationships. The use of convenience sampling and the study's sample size may limit its representativeness of the broader Saudi population, which affects the generalizability of the results. Given that the study relied on self-reported online survey data, recall bias and social desirability bias may have affected its accuracy and reliability. Future research could explore regional variations in knowledge and perceptions and investigate the effectiveness of specific educational interventions. To mitigate these limitations in future research, a randomized or stratified sampling approach could be utilized to ensure a more representative sample. Longitudinal studies could help track changes in knowledge and attitudes over time, providing deeper insights into the effectiveness of awareness campaigns and medical interventions. Furthermore, incorporating qualitative methods, such as structured interviews or focus groups, would allow for a richer exploration of participants’ perspectives and reduce the risk of misinterpretation associated with self-reported surveys.

## Conclusions

The study revealed significant gaps in public knowledge and perception regarding radiation exposure risks among the participants. Receiving information about radiation exposure risk was a strong predictor of increased knowledge and awareness. A gender difference in perceptions was observed, with males and married participants being less likely to hold positive perceptions of radiation exposure risks. The findings highlight the need for targeted educational interventions to address knowledge gaps and misconceptions and raise awareness. Additionally, tailoring communication strategies to individual experiences and concerns during radiological procedures is crucial for fostering patient trust and encouraging a change in attitudes.
